# Anti-Tumoral Effect of Chemerin on Ovarian Cancer Cell Lines Mediated by Activation of Interferon Alpha Response

**DOI:** 10.3390/cancers14174108

**Published:** 2022-08-25

**Authors:** Meike Schmitt, Johanna Gallistl, Susanne Schüler-Toprak, Jürgen Fritsch, Christa Buechler, Olaf Ortmann, Oliver Treeck

**Affiliations:** 1 Department of Gynecology and Obstetrics, University Medical Center Regensburg, 93053 Regensburg, Germany; 2 Department of Infection Prevention and Infectious Diseases, University Medical Center Regensburg, 93053 Regensburg, Germany; 3 Department of Internal Medicine I, University Medical Center Regensburg, 93053 Regensburg, Germany

**Keywords:** ovarian cancer, cell line, chemerin, adipokine, interferon alpha

## Abstract

**Simple Summary:**

Chemerin is a multifunctional protein with an important role in the immune system. Recent evidence showed that chemerin also regulates the development of cancer. Ovarian cancer is a common type of tumor in women. In this study, we observed that chemerin decreases the growth of ovarian cancer cell lines in vitro when cultivated in standard cell culture or in globular multicellular aggregates. When we examined the mechanisms involved in this process, we found that treatment of ovarian cancer cells with chemerin led to the activation of genes that are known to mediate the effects of interferon alpha (IFNα). The main effect of IFNα is to defend body cells against viral infections, but it is also able to defeat cancer cells. We observed that this activation of IFNα response by chemerin resulted from the increased production of IFNα protein in ovarian cancer cells, which then reduced cancer cells numbers. However, it remains to be investigated how exactly chemerin might be able to activate interferon alpha and its anti-tumoral actions.

**Abstract:**

The pleiotropic adipokine chemerin affects tumor growth primarily as anti-tumoral chemoattractant inducing immunocyte recruitment. However, little is known about its effect on ovarian adenocarcinoma. In this study, we examined chemerin actions on ovarian cancer cell lines in vitro and intended to elucidate involved cell signaling mechanisms. Employing three ovarian cancer cell lines, we observed differentially pronounced effects of this adipokine. Treatment with chemerin (huChem-157) significantly reduced OVCAR-3 cell numbers (by 40.8% on day 6) and decreased the colony and spheroid growth of these cells by half. The spheroid size of SK-OV-3 ovarian cancer cells was also significantly reduced upon treatment. Transcriptome analyses of chemerin-treated cells revealed the most notably induced genes to be interferon alpha (IFNα)-response genes like IFI27, OAS1 and IFIT1 and their upstream regulator IRF9 in all cell lines tested. Finally, we found this adipokine to elevate IFNα levels about fourfold in culture medium of the employed cell lines. In conclusion, our data for the first time demonstrate IFNα as a mediator of chemerin action in vitro. The observed anti-tumoral effect of chemerin on ovarian cancer cells in vitro was mediated by the notable activation of IFNα response genes, resulting from the chemerin-triggered increase of secreted levels of this cytokine.

## 1. Introduction

Ovarian cancer is the fifth most common cause of death because of cancer in women and is the leading cause of death from gynecological malignancies in the developed world [1]. As the deadliest gynecological malignancy, it has a 5-year survival rate of only 10%, when the most common serous type spreads rapidly throughout the peritoneal cavity. Overall, this disease has a poor prognosis with a 5-year survival rate of approximately 50%. If diagnosed in earlier stages when the cancer is still confined to the ovary, this survival rate could rise to about 90%, but today this occurs in only 20% of patients [2,3]. Ovarian cancer includes a heterogeneous group of neoplasias, and among this group, about 90% are of epithelial origin (mucinous, serous, endometrioid, and clear cell subtypes) [4]. Most of the women are diagnosed with high-grade serous ovarian cancer (HGSOC), more aggressive than the low-grade serous ovarian cancer and characterized by a poor prognosis [5,6]. Increasing evidence suggests that ovarian cancer, like tumors of different origins, is affected by the adipokine chemerin [7,8,9,10,11,12,13,14,15]. Local and circulating levels of chemerin are positively correlated with BMI and obesity-related biomarkers since it is primarily secreted from white adipose tissues [16], later studies also showed liver and lung as further sources of this adipokine. Chemerin, coded by the gene RARRES2, is a multifunctional protein with established roles as an chemoattractant in inflammation, as an inductor of adipogenesis and as an important factor in glucose homeostasis [17,18,19,20]. However, its role in cancer is only beginning to be understood and is able to exert both anti-tumoral and tumor-promoting effects, depending on the cancer type and its mode of action (which we reviewed in [15]). Chemerin is synthesized as a 163 aa inactive precursor that undergoes proteolytic processing at both termini, resulting in different chemerin variants. For this study, we used the bioactive recombinant huChem-157, a 16 kDa protein (aa 21–157) (also referred to as chemerin in the results and discussion section) [21]. Chemerin signaling is mediated by its binding to the receptors chemokine-like receptor 1 (CMKLR1) and G-protein coupled receptor 1 (GPR1) [22,23]. C-C chemokine receptor-like 2 (CCRL2) is a non-signaling receptor proposed to present chemerin at the cell surface [24]. Chemerin receptors are expressed in immune cells and adipocytes, and to a smaller degree in most normal and tumor tissues [25]. With regard to non-tumorigenic ovarian tissue like in polycystic ovary syndrome (PCOS), a disorder linked to low-grade chronic inflammation, elevated blood levels of inflammatory cytokines and adipokines like chemerin were reported [26], and in these patients, a significant association between high intrafollicular androgen levels and chemerin [27] was reported. In granulosa cells (GC), bioactive chemerin and its receptor CMKLR1 have been detected [28], and PCOS patients had elevated levels of chemerin in GC associated with local insulin resistance [29]. Using an obesity mouse model, the chemerin/CMKLR1 system was observed to be up-regulated in blood, ovaries, and granulosa cells and was associated with apoptotic ovarian follicles, oxidative stress, and apoptosis biomarkers. Further in vitro experiments confirmed the apoptotic effect of chemerin on granulosa cells [30].

With regard to cancer, chemerin is thought to affect tumor development via three main mechanisms: (1) suppressing the tumor as a strong chemoattractant inducing immunocyte recruitment to the tumor, (2) promoting the tumor by activating endothelial angiogenesis, and (3) affecting the intracellular signaling of tumor cells via CMKLR1 and GPR1, resulting in context-dependent anti-tumoral or tumor-promoting actions (reviewed in [15]); [10,12,15,31,32,33]. In ovarian cancer, high levels of active chemerin have been found in a large proportion of the ascitic fluids of ovarian carcinomas [34]. A recent study did not observe effects of chemerin on proliferation of ovarian cancer cells but reported chemerin to inhibit bisphenol A-induced ovarian tumor cell growth [35]. With regard to the expression of chemerin and its receptors in ovarian cancer and their association with survival, we recently analyzed open-source DNA microarray and survival data of 1656 ovarian cancer patients using the KM-Plotter platform (https://kmplot.com/analysis/index.php?p=service&cancer=ovar (accessed on 18 December 2021)) and identified a slightly lower chemerin expression in ovarian cancer tissue than in normal ovaries (*p* = 0.018) [36]. However, these tissues are only minor sources of this adipokine, which is predominantly secreted from white adipose tissue. Importantly, higher intratumoral expression of CMKLR1 has a beneficial effect both on overall survival (OS) (*p* = 0.002, HR = 0.8 (0.7–0.92)) and on progression-free survival (PFS) (*p* = 0.026, HR = 0.86 (0.76–0.98) in ovarian cancer patients [36].

Given that only limited insight exists regarding the effects of chemerin on ovarian cancer cells and particularly on intracellular signaling, we performed an in vitro study of three ovarian cancer cell lines. We examined the effects of bioactive chemerin (huChem-157) on their growth, apoptosis, migration, and invasion and analyzed molecular mechanisms underlying the action of this adipokine by means of transcriptome and pathway analyses.

## 2. Materials and Methods

### 2.1. Materials

OVCAR-3, OAW-42 and SK-OV-3 ovarian cancer cells were obtained from American Type Culture Collection (Manassas, VA, USA). Recombinant huChem-157 was from R&D Systems (Minneapolis, MN, USA), (Cat. No. 2324- CM-025), which is the 16 kDa processed monomeric form of chemerin being most bioactive in the human body (aa 21–157). DMEM/F12 culture medium, FBS, sodium pyruvate, insulin, L-glutamine and Accutase were obtained from Sigma-Aldrich (Munich, Germany). Affinity Script Multi Temperature cDNA Synthesis Kit was from Agilent (Santa Clara, CA, USA). RNeasy Mini Kit, RNase Free DNase Set and Quantitect SYBR Green PCR Kit were obtained from Qiagen (Hilden, Germany). PCR primers were synthesized at Eurofins (Hamburg, Germany).

### 2.2. RNA Preparation and Real-Time RT-PCR

Total RNA from cell lines was isolated using RNeasy Micro Kit (Qiagen), RNA was then reversely transcribed using the Affinity Script Multi Temperature cDNA Synthesis Kit (Invitrogen, Karlsruhe, Germany) according to manufacturer’s protocols. For qPCR analysis, 4 µL of cDNA were amplified using LightCycler^®^ FastStart DNA MasterPLUS SYBR Green I (Roche Diagnostics GmbH, Mannheim, Germany) and 5 mM of each primer in a LightCycler^®^ 2.0 Instrument (Roche, Mannheim, Germany) under the following conditions: initial denaturation at 95 °C for 15 min, followed by 35 cycles of 10 s denaturation at 95 °C, 5 s annealing at 60 °C and 12 s extension at 72 °C. Data were analyzed using the comparative ΔΔCT method calculating the difference between the threshold cycle (CT) values of the target and reference gene of each sample and then comparing the resulting ΔCT values between different samples [37,38]. Primer sequences were:

IFI44:  5′-GCGGCCTGTGCAGGGATGAC-3′ and 5′-TGTCCTTCAGCGATGGGGAATCA-3′,
OAS1:    5′-GAGGCAGCTGGCACAAGAGGC-3′ and 5′-CGTCGGTCTCATCGTCTGCAC-3′,
DDX60:  5′-CGCGGGTCTTTGGACACCACC-3′ and 5′-GCTGCCTGTGCCTCCAACCTG-3′,
MX1:      5′-GGCTGTTTACCAGACTCCGACA-3′ and 5′-CACAAAGCCTGGCAGCTCTCTA-3′
ACTB:    5′-CACCATTGGCAATGAGCGGTTC-3′ and 5′-AGGTCTTTGCGGATGTCCACGT-3′

### 2.3. Cell Culture, Cell Viability, Soft Agar Colony and Spheroid Formation

OVCAR-3, OAW-42 and SK-OV3 ovarian cancer cells were maintained in DMEM-F12 medium containing FBS and were cultured with 5% CO_2_ at 37 °C in a humidified incubator. OVCAR-3 cells were supplemented with 0.01 mg/mL bovine insulin and 20% FBS. For the cell viability assays, 1000 cells were seeded per well in a 96-well plate, and after adhesion, treatment with 0, 100 or 400 ng/mL chemerin followed. Cells were cultivated for 6 days, and on days 0, 3, 4, 5 and 6, relative numbers of viable cells were measured with the fluorometric, resazurin-based Cell Titer Blue (CTB) assay (Promega, Madison, WI, USA), at 560Ex/590Em nm in a Victor3 multilabel counter (PerkinElmer, Rodgau, Germany).

For the soft agar colony formation assays, 5 mL DMEM containing 10% FBS and 0.75% melted agar were mixed and put into a 60 mm culture dish as a bottom layer. After this layer had become solid at RT, 10^4^ cells (treated with 0, 100 or 400 ng/mL chemerin) in 3 mL of DMEM containing 10% FBS and 0.36 mL melted agar were prepared and slowly pipetted onto the bottom layer. The culture dishes were then incubated at 37 °C in a humidified incubator for 3 weeks. During this time, once a week, fresh culture medium including chemerin was added. After this, the colonies were counted and stained with 0.04% crystal violet/2% ethanol in PBS, and photographs were taken. Additionally, we compared median colony sizes of unstained colonies using a bright-field microscope with 50× magnification. From the taken photographs, the size of each of the 50 colonies was analyzed by means of ImageJ software (NIH).

For the generation of spheroids, we used a relatively new but approved method: the ultra-low attachment “5D Sphericalplates” (SP5D, Kugelmeiers, Erlenbach, Switzerland, www.sp5d.com) [39,40,41,42]. The SP5D is a 24-well plate with 750 microwells per well in 12 functionalized wells, allowing the formation of 9000 spheroids per plate. A nanocoating facilitates cell aggregation in microwells. Before cell seeding, the functionalized wells (containing microwells) of the SP5D were rinsed using 1 mL culture medium. Then, 0.5 mL suspension containing the calculated number of cells (300 cells per microwell) was prepared and sieved through a 70 µm cell strainer to obtain a single-cell suspension without clusters. After that, 0.5 mL cell-free DMEM-F12 with 10% FBS was added per well, and the plate was tapped to remove air bubbles. This single-cell suspension was added to these wells for a total of 1 mL per well, and the plates were then cultured under standard conditions. The next day, the cells were treated with chemerin (final medium concentration 400 ng/mL) or medium as control. Five days after treatment, photographs of each plates that showed more than 50 grown spheroids were taken using bright field microscopy. From these, the sizes of the 50 spheroids per condition and cell line were determined using ImageJ software (NIH, Bethesda, MD, USA).

### 2.4. Apoptosis Assays

To examine whether cellular apoptosis was induced in the ovarian cancer cell lines after treatment with chemerin, we used two different approaches. First, we performed Western blot experiments with cell lysates of cells treated with chemerin for 48 h and used antibodies able to detect PARP1-cleavage and cleavages of caspases 3 and 8 as described in 2.7. Second, we performed a FACS analysis of the chemerin-treated cells (400 ng/mL, 48 h) using the Guava^®^ Muse^®^ Cell Analyzer (Luminex, Austin, TX, USA), using the Muse^®^ Annexin V & Dead Cell Kit following the instructions of the manufacturer. This method allowed for the detection of cells in early apoptosis, late apoptosis and non-apoptosis.

### 2.5. Invasion and Migration Assay

The modified Boyden chamber model was employed to examine chemerin’s effects on cellular invasion and migration using the Cultrex Basement Membrane Extract Cell Invasion Assay, 24-well (R&D Systems). Prior to seeding the employed cell lines in the upper chamber of the assay, the cells being cultured in DMEM/F12 with 10% FBS supplemented with 2 mM L-glutamine, and in the case of OVCAR-3 cells additionally with 0.01 mg/mL bovine insulin, were serum starved by cell culture in 1% FBS and 0.5× SR2 (Serum replacement 2, Merck (Kenilworth, NJ, USA)/Sigma Aldrich) (including the supplements mentioned above) for 24 h. Then, cells were cultured in DMEM/F12 with 1× SR2 (plus supplements) for another day. The invasion and migration assays were performed according to the manufacturer’s protocol. In brief, for the invasion studies, the in vitro invasion chamber model was prepared by adding 100 µL ice-cold liquid BME (basement membrane extract) to the pre-cooled insert followed by placing the BME-coated plate at 37 °C overnight in a cell culture incubator, allowing for BME polymerization. The next day, 200 µL suspension containing 50,000 serum-starved cells +/− chemerin was seeded per well into the BME-coated insert. After 600 µL culture medium containing 10% FBS were filled into the lower chamber, the BME-coated upper chamber seeded with cells was inserted into the bottom chamber and the whole plate was placed in a CO_2_ incubator at 37 °C. On days 2, 4 and 6 after starting the assay, the presence of invaded cells in the bottom chamber was checked microscopically. When more than 100 cells were observed at the base of this chamber, the bottom side of the insert was treated with trypsin to detach the remaining invaded cells, which were then added to the cells in the bottom chamber. To determine the total number of invaded cells, the CTB assay was used and measured fluorometrically using the Victor3 counter (PerkinElmer). For the examination of cellular migration, the same method was used but in the absence of BME coating. As an internal control, a bottom chamber without chemoattractant FBS was used.

### 2.6. Affymetrix Clariom S Microarray Assay

The RNA samples were prepared for microarray hybridization as described in the Affymetrix GeneChip WT PLUS Reagent Kit User Manual (Affymetrix, Inc., Santa Clara, CA, USA). Labeled ss cDNA was hybridized to Affymetrix Clariom S human arrays for 16 h at 45 °C and 60 rpm in a GeneChip hybridization oven 640. Hybridized arrays were washed and stained in an Affymetrix Fluidics Station FS450, and the fluorescent signals were measured with an Affymetrix GeneChip Scanner 3000 7G. Sample processing was performed at an Affymetrix Service Provider and Core Facility, “KFB—Center of Excellence for Fluorescent Bioanalytics” (Regensburg, Germany; www.kfb-regensburg.de). Microarray data analysis and statistics were performed as described previously [43].

### 2.7. Western Blot Analysis

For the preparation of cell lysate, cells were lysed in RIPA buffer (1% (*v/v*) Igepal CA-630, 0.5% (*w/v*) sodium deoxycholate, 0.1% (*w/v*) sodium dodecyl sulphate (SDS) in phosphate-buffered solution (PBS) containing aprotinin and sodium orthovanadate. Aliquots of cell lysate containing 10 µg of protein were resolved by 10% (*w/v*) SDS–polyacrylamide gel electrophoresis, followed by electrotransfer to a PVDF hybond (Amersham, UK) membrane. Immunodetection was carried out using CMKLR1 antibody (Abcam, Cambridge, UK, ab64881, 1:500), GPR1 antibody (antibodies-online, ABIN516152, 1:200), IRF9 antibody (Abcam, ab282125, 1:1000), MX1 antibody (Abnova H00004599-D01P, 1:1000), anti-PARP (#9542 Cell Signaling, Danvers, MA, USA; 1:1000), anti-cleaved Caspase-3 (#9661 Cell Signaling; 1:1000), anti-Caspase-8 (ALX-804-242-C100 ENZO Lifesciences; 1:1000), anti-b-Actin-HRP (HRP-60008 Proteintech; 1:30,000), anti-rabbit-HRP (111-035-144 Jackson Immuno Research; 1:10,000) and β-actin antibody (1:500) (ab8226, Abcam), which were detected using chemiluminescence (ECL) system (Amersham, Buckinghamshire, UK).

### 2.8. Statistical Analysis

Statistical analysis was performed by means of Student´s *t*-test or the nonparametric Kruskal–Wallis test with Dunn´s posttest. For statistics, we used Graph Pad Prism Version 7.04 Software (Graph Pad, San Diego, CA, USA). Statistical significance was set at *p* value lower than 0.05.

## 3. Results

### 3.1. Expression of Chemerin Receptors CMKLR1 and GPR1 in Ovarian Cancer Cell Lines

**Figure 1:**
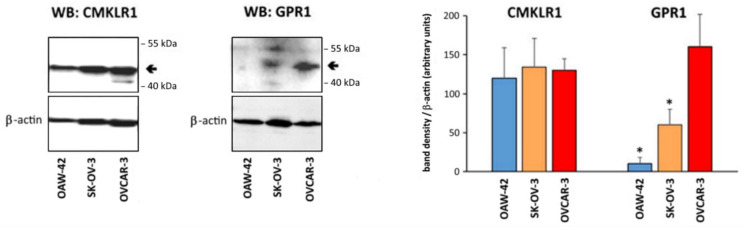
Western blot analysis of the chemerin receptors CMKLR1 and GPR1 in ovarian cancer cell lines employed in this study. As reference, β-actin levels were detected. For preparation of cell lysates, cells were lysed in RIPA buffer and aliquots containing 10 µg protein were resolved by SDS–PAGE, followed by electrotransfer to a PVDF membrane. Antibodies used were anti-CMKLR1 (Abcam, ab64881, 1:500), anti-GPR1 (antibodies-online, ABIN516152, 1:200), and anti-ACTB (Abcam, ab8226, 1:500) and a horseradish peroxidase conjugated secondary antibody (1:20,000) which was detected using ECL system as described in the methods section. Western blot results from three independent experiments were densitometrically analyzed (ImageJ software, NIH). Shown are representative Western blots (left) and diagrams of mean band density (*n* = 3). * *p* < 0.001 vs. OVCAR-3. Full pictures of the Western blots are presented in File S1.

First, we characterized the employed ovarian cancer cell lines OVCAR-3, OAW-42 and SK-OV-3 with regard to their expression of the receptors CMKLR1 and GPR1, which are known (unlike CCRL2) to affect intracellular signaling and thus are relevant for this in vitro study. For this purpose, we examined their expression at the protein level using Western blot analysis (Figure 1). Whereas the expected CMKLR1 band appeared in all tested cell lines to a similar degree, protein expression of GPR1 was most pronounced in OVCAR-3 cells, medium in SK-OV-3 and very low or absent in OAW-42 cells.

### 3.2. Effect of Chemerin (huChem-157) on Ovarian Cancer Cell Lines Using 2D and 3D In Vitro Culture Models

**Figure 2:**
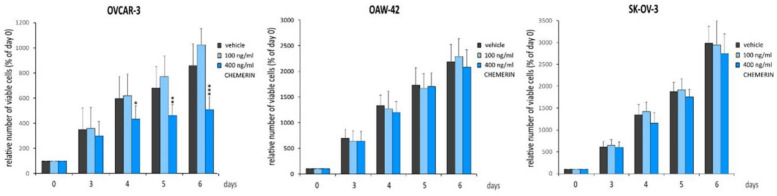
Effects of chemerin (recombinant huChem-157) (0, 100 and 400 ng/mL) on relative cell numbers of the indicated ovarian cancer cell lines as assessed by means of the fluorometric Cell Titer Blue assay (Promega). Cells were treated on day 0 and cultured for 6 days in medium supplemented with 10% FBS (*n* = 4). * *p* < 0.05; ** *p* < 0.001; *** *p* < 0.0001 vs. vehicle control.

We first examined the effect of bioactive recombinant human chemerin (huChem-157) (R&D Systems, 2324-CM-025) on ovarian cancer cell lines in standard adherent 2D culture conditions. To choose the concentrations for treatment, we considered a study reporting that 100 ng/mL of huChem-157 is equivalent to a concentration of 25 ng/mL chemerin using standard ELISA [44]. Thus, we used 100 and 400 ng/mL of huChem-157, being equivalent to 25 or 100 ng/mL serum chemerin, which represents the usual range from non-obese to obese patients in vivo [45]. Three ovarian cancer lines, the sex steroid hormone-responsive lines OVCAR-3 and OAW-42 and the hormone-unresponsive, HER-2 overexpressing line SK-OV-3, were treated with 0, 100 and 400 ng/mL of huChem-157. Chemerin’s effects on these cells in vitro were measured with the fluorometric Cell Titer Blue Assay (Promega), which allows for the determination of relative amounts of viable cells. Treatment with the higher chemerin concentration (400 ng/mL) significantly inhibited the number of viable OVCAR-3 cells in a time-dependent manner after 4, 5 and 6 days in culture (Figure 2). When compared with the negative control, cell numbers were found to be decreased by 27.1% on day 4 (*p* < 0.05), by 32.1% on day 5 (*p* < 0.001) and by 40.8% on day 6 (*p* < 0.0001). In contrast, treatment with the lower chemerin concentration (100 ng/mL) did not show significant changes in OVCAR-3 cell numbers but did show an increasing trend. With regard to OAW-42 and SK-OV-3 cells, none of the chemerin doses tested resulted in significant effects, but in SK-OV-3 cells, treatment with 400 ng/mL chemerin resulted in a trend towards decreasing cell numbers beginning at 4 days of treatment.

**Figure 3:**
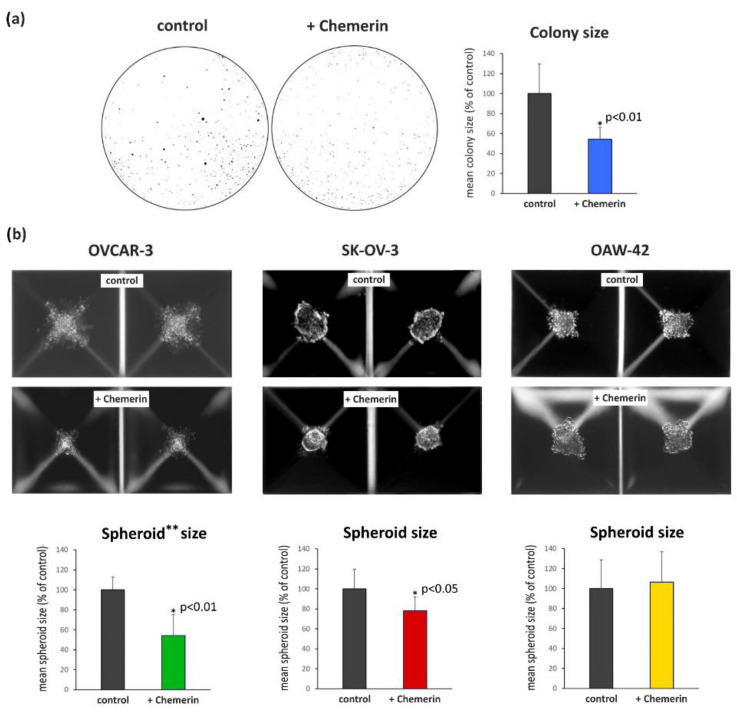
(**a**) Effects of chemerin (huChem-157) (0 and 400 ng/mL) on the colony sizes of OVCAR-3 cells grown for three weeks in soft agar. Colony numbers were not significantly affected. Cells were treated with chemerin once a week. (*n* = 3). * *p* < 0.01 vs. control. (**b**) Effects of chemerin (huChem-157) (0 and 400 ng/mL) on growth of spheroids of the indicated cell lines, which displayed a specific phenotype and density, generated using “5D Sphericalplates” (tebu-bio, Kugelmeiers, Erlenbach, Switzerland). Upper panel: Photomicrographs of the spheroids or clusters grown in these plates generated by means of bright field microscopy. Bottom panel: For determination of the mean size of the spheroids or clusters, 50 per cell line were measured using ImageJ software (NIH). ** Spheroid generation of this cell line resulted in loose, irregular formed cell clusters.

Next, we performed colony formation assays to examine the ability of chemerin to affect the anchorage-independent growth of the employed ovarian cancer cell lines. We only observed an effect of 400 ng/mL on mean colony sizes of OVCAR-3 cells grown for 3 weeks in soft agar (Figure 3a) but not on SK-OV-3 cells, whereas OAW-42 cells did not form colonies in soft agar. In contrast, colony numbers did not significantly change after chemerin treatment in any cell lines tested (data not shown). Further experiments examining whether the size of 3D spheroids grown from these cell lines would be affected by chemerin showed that spheroid formation was cell-line dependent, resulting in dense SK-OV-3 spheroids, OAW-42 spheroids with a medium density and irregular shape and scattered OVCAR-3 cells aggregates. Chemerin treatment (400 ng/mL) reduced the growth of the loose 3D aggregates of OVCAR-3 cells by 46% (*p* < 0.01) and the mean size of the SK-OV-3 spheroids by 21.5% (*p* < 0.05). In contrast, no chemerin effect on spheroid size was observed in OAW-42 cells (Figure 3b).

### 3.3. Effect of Chemerin on Apoptosis of Ovarian Cancer Cell Lines

**Figure 4:**
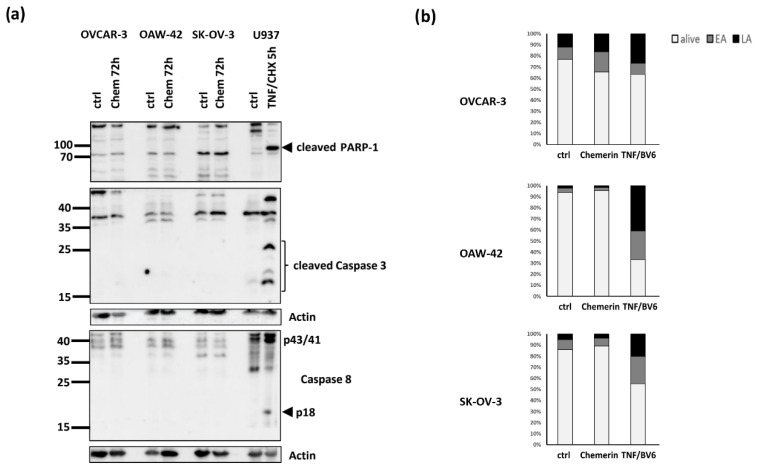
Effects of chemerin (huChem-157) (0 and 400 ng/mL) treatment for 48 h on cellular apoptosis. (**a**) Western blot analysis of PARP-1 cleavage, cleaved caspase 3 and caspase 8. Cell lysate from U937 cells treated with TNF/CHX (cycloheximide) (100 ng/mL; 0.5 µg/mL) was loaded as positive control. (**b**) FACS analysis by means of (Guava^®^ Muse^®^ Cell Analyzer, Luminex, Austin, TX, USA), using the Muse^®^ Annexin V & Dead Cell Kit. The indicated cell lines were treated with chemerin (400 ng/mL) for 48 h or as a positive control with TNF/BV6 (100 ng/mL; 2.5 µM). EA = early apoptosis, LA = late apoptosis.

Next, we examined to what extent treatment with chemerin would trigger apoptosis in the employed cell lines. For this purpose, we first performed Western blot analyses of PARP-1 cleavage and cleaved caspases 3 and 8 (Figure 4a). Treatment with chemerin (400 ng/mL) did not induce cleavage of PARP-1, caspase 3 or caspase 8, indicating that this adipokine did not trigger apoptosis in the employed cell lines. The cleavage of these proteins was only observed in the positive control, a combination of TNF and cycloheximide (CHX). By means of flow cytometry (Guava^®^ Muse^®^ Cell Analyzer, Luminex, Austin, TX, USA), using the Muse^®^ Annexin V & Dead Cell Kit, we observed no significant apoptosis 48 h after treatment. OVCAR-3 cells exhibited a slight increase in early and late apoptosis that was only pronounced in the positive control, a combination of TNF and BV6 (the latter being an antagonist of IAP inhibitor of apoptosis proteins) (Figure 4b), (Supplementary Figure S1). Microphotographs of the ovarian cell lines did not show any change of cell morphology after treatment with chemerin, in contrast to the positive control treatment resulting in visible cell damage (Supplementary Figure S2).

### 3.4. Effect of Chemerin on Migration and Invasiveness of Ovarian Cancer Cell Lines

OVCAR-3, SK-OV3 and OAW-42 ovarian cancer cells were reported to have invasive properties as assessed in vitro with the penetration of a reconstituted basement membrane [46,47]. We examined to what extent treatment with chemerin would affect the invasiveness of these cell lines in vitro using a modified Boyden-chamber model coated with basement membrane extract (BME) (Cultrex BME Cell Invasion Assay (R&D Systems, Minneapolis, MN, USA). All cell lines tested were able to invade and cross the artificial basement membrane. Neither OVCAR-3, OAW-42 nor SK-OV-3 cells treated with chemerin (400 ng/mL) exhibited an altered invasiveness. Examining to what extent chemerin would affect cellular migration using the same assay without basement membrane coating also did not reveal any chemerin effects on the employed ovarian cancer cell lines, and neither did wound-healing scratch assays (data not shown).

### 3.5. Chemerin Effects on Transcriptomes of Ovarian Cancer Cell Lines

**Figure 5:**
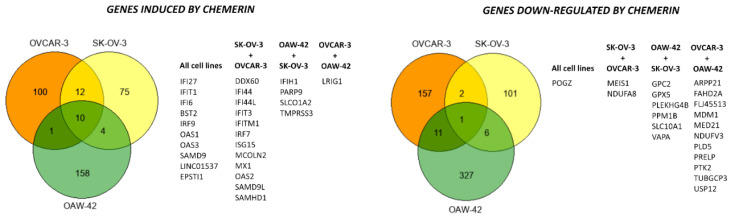
Effects of chemerin (huChem-157) (400 ng/mL) on the transcriptome of the indicated ovarian cancer cell lines as assessed by means of Affymetrix Clariom S human arrays. Cells were treated with chemerin for 48 h, and isolated RNA was processed and hybridized as described in the methods section. Venn diagrams show the number of genes with at least 2.5-fold change in mRNA expression.

To elucidate the molecular mechanisms underlying the observed effect of chemerin (huChem-157) on the growth of OVCAR-3 and SK-OV-3 cells using 2D and 3D culture models, we examined the transcriptome changes triggered by this adipokine using Human Affymetrix Clariom S DNA microarrays. For comparison, we also analyzed the transcriptome of OAW-42 cells that were not responsive to chemerin-triggered growth reduction. Considering genes exhibiting an at least 2.5-fold change in mRNA levels 48 h after chemerin treatment, 123 genes were up-regulated in OVCAR-3 cells, 173 genes had an increased transcript level in OAW-42 cells and 101 genes were induced on the mRNA level in SK-OV-3 cells. Whereas the transcript levels of many of these genes were altered in a cell-line specific manner, we found ten genes to be up-regulated in all cell lines tested, demonstrating the presence of common effects on ovarian cancer cells (Figure 5, left panel). Twelve genes exhibited increased mRNA levels both in SK-OV-3 and OVCAR-3 cells, whereas four genes were up-regulated in both OAW-42 and SK-OV-3 cells. Subsequent pathway analyses using Ingenuity Pathway Analysis (IPA) software (Qiagen) revealed most of these induced genes to be type I interferon response genes. With regard to genes with decreased transcript levels 48 h after chemerin treatment, only POGZ gene, coding for the protein “Pogo transposable element with ZNF domain” was affected in all cell lines tested, whereas hormone-dependent OVCAR-3 and OAW-42 cells shared the highest number of down-regulated genes, including the tumor-promoting genes PTK2 and USP12 (Figure 5, right panel). Genes exhibiting the strongest changes in transcript levels are listed in Supplementary Table S1.

#### 3.5.1. Verification of the Microarray Data by RT-qPCR and Western Blot Analyses

**Figure 6:**
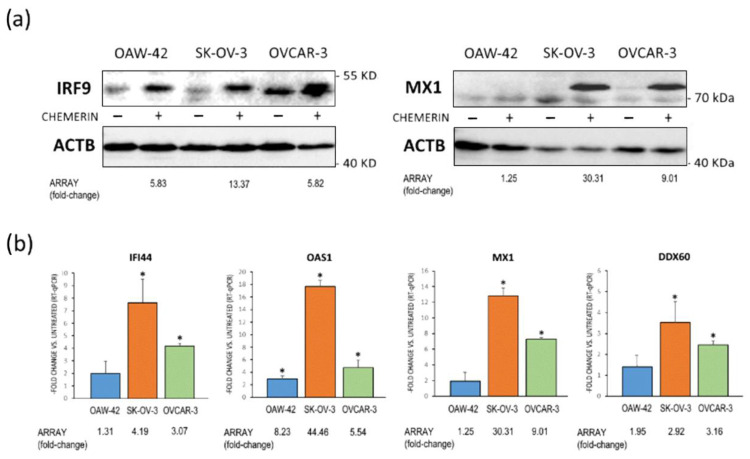
Verification of the Affymetrix DNA microarray data. Expression of the indicated genes induced upon chemerin (huChem-157) (400 ng/mL) treatment was analyzed by Western blot or RT-qPCR. Shown is their expression in the indicated ovarian cancer cell lines in (**a**) representative Western blots or (**b**) in fold-change of mRNA levels normalized to β-actin (ACTB) expression compared to untreated cells (RT-qPCR). For comparison, the -fold change values after chemerin treatment assessed by microarray analyses are indicated. * *p* < 0.01 (*n* = 3). Full pictures of the Western blots are presented in File S1.

Next, we performed validation experiments to confirm the DNA microarray results by testing the effects of chemerin on the expression of selected genes on the protein and mRNA levels. Using Western blot analysis, we examined the protein expression of IRF9 gene, the transcript level of which was elevated upon chemerin treatment in all cell lines tested, and MX1 gene, showing increased mRNA expression after chemerin treatment in SK-OV-3 and OVCAR-3 cells only. The results of these experiments demonstrated that chemerin affected not only transcript but also the protein levels of these genes to a similar extent (Figure 6a). The microarray-based mRNA data were then exemplarily verified by means of RT-qPCR. Examining the expression of IFI44 gene with this method confirmed the microarray data showing increased mRNA levels in SK-OV-3 and OVCAR-3 but not in OAW-42 cells, and the analysis of gene OAS1 demonstrated that its transcript levels were strongly elevated in all cell lines tested in both the microarray and RT-qPCR experiments, with a particularly pronounced increase in SK-OV-3 cells. Transcript levels of MX1 gene were confirmed to be up-regulated after chemerin treatment in OVCAR-3, and to a higher extent in SK-OV-3 cells, whereas mRNA expression of DDX60 gene was verified as significantly elevated in SK-OV-3 and OVCAR-3 cells (Figure 6b). Thus, the analysis of the selected genes verified the regulation trends revealed by our microarray experiments.

#### 3.5.2. Gene and Pathway Analyses Based on the Chemerin-Triggered Transcriptome Changes

**Figure 7:**
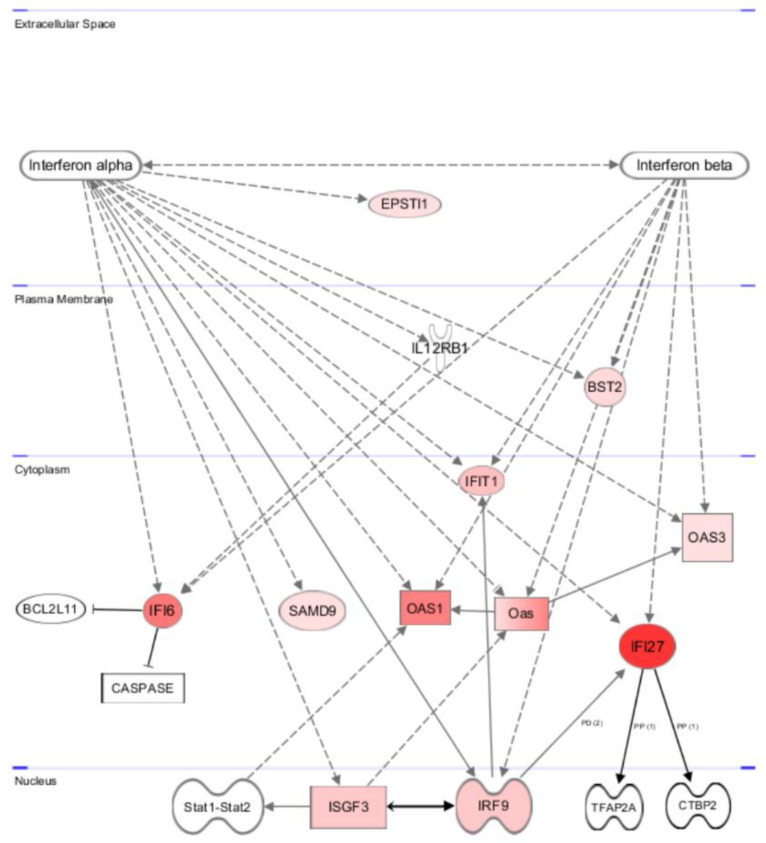
Pathway analysis of the genes being up-regulated in all chemerin (huChem-157)-treated ovarian cancer cell lines after 48 h as assessed with Affymetrix Clariom S human microarrays. All genes with elevated transcript levels (red) are type I interferon response genes, mostly induced by IFNα-triggered assembly of upstream regulator and transcription factor complex ISGF3, consisting of IRF9 and STAT1/2 (ISGF3 effects are not shown here). Also indicated is the cellular localization of the gene products. Broken arrows indicate transcriptional activation, other arrows protein interaction. The intensity of red color represents the measured grade of transcript up-regulation after chemerin treatment (Ingenuity pathway analysis (IPA) software, Qiagen).

Using Ingenuity Pathway Analysis software (IPA, Qiagen), we first analyzed the 10 genes that were induced upon chemerin treatment in all cell lines tested, namely IFI27, IFIT1, IFI6, BST2, IRF9, OAS1, OAS3, SAMD9, LINC01537 and EPSTI1. Nine out of these ten genes turned out to be target genes of interferon alpha (IFNα), and seven also were known IFNα responsive genes (Figure 7). Binding type I interferons to the receptor IFNAR1/2 activates Tyk2 and JAK1 and triggers the dimerization of STAT1 and STAT2, which bind to IRF9, forming the interferon-stimulated gene factor 3 (ISGF3) transcription complex. ISGF3 complex enters the nucleus and activates the transcription of a variety of interferon-responsive genes by binding to *interferon-stimulated response elements* (ISREs), resulting in the activation of immune response and other processes [48].

**Figure 8:**
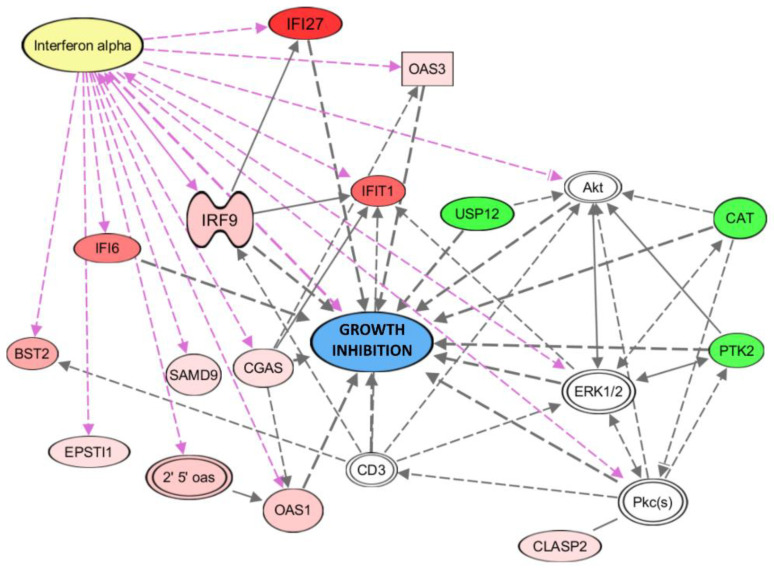
Pathway analysis of key genes being up- or down-regulated on the transcript level upon 48 h of chemerin (huChem-157) treatment in OVCAR-3 cells as assessed by Affymetrix Clariom S human microarrays. Most of the up-regulated genes (red) are IFNα-response genes, with IRF9 as the key mediator of IFNα-induced gene regulation (as part of the ISGF3 complex, not shown). The observed regulation pattern is predicted to lead to growth inhibition of tumor cells (Ingenuity pathway analysis (IPA) software, Qiagen) (see discussion section). Broken arrows indicate transcriptional activation, other arrows protein interaction. The color intensity represents the measured grade of mRNA up- (red) or down-regulation (green).

Given that the chemerin effects on 2D and 3D growth were most pronounced in OVCAR-3 cells, we next analyzed the transcriptome changes triggered by chemerin in this cell line only. Pathway analysis software (IPA, Qiagen) revealed a network of up- and down-regulated genes, and their regulation pattern was identified to exert anti-tumoral, growth-inhibitory effects (Figure 8) (details in discussion section). Among the most prominent up-regulated genes were IFNα response genes, with IFI27, IFIT1 and IFI6 exhibiting the strongest induction. On the other hand, the identified genes with notable decreased mRNA levels playing roles in tumor growth promotion were USP12, CAT and PTK2 (FAK). Interferon regulatory factor 9 (IRF9), itself responsive to IFNα, exhibited 5.8-fold up-regulated transcript levels, and was identified to be the key mediator of IFNα action, as IRF9 protein as part of the ISGF3 transcription factor complex is known to induce responsive genes and to exert anti-tumoral actions by binding to interferon-sensitive response elements (ISREs) in their regulatory region [49,50].

**Figure 9:**
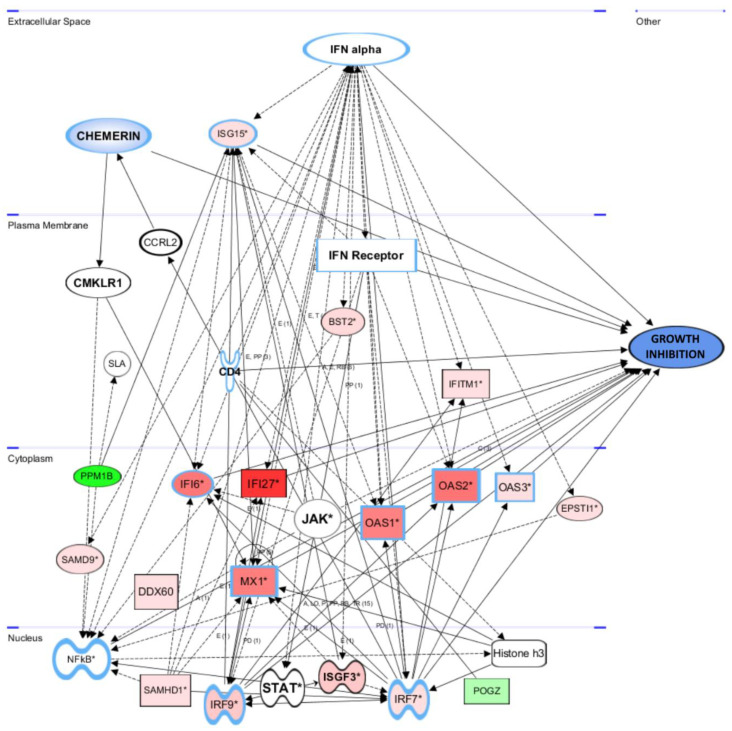
Network of genes regulated after 48 h of chemerin (huChem-157)-treatment in at least two ovarian cancer cell lines, and connection to tumor growth inhibition. Activation of the network of interferon response genes is mediated by IFNα-induced formation of the upstream regulator and transcription factor ISGF3 (ISGF3 target genes are indicated by an asterisk). Broken arrows indicate transcriptional activation, other arrows protein interaction. Connection of genes to growth inhibition is indicated using solid arrows. The color intensity represents the measured grade of mRNA up- (red) or –down-regulation (green). * Type I interferon response genes (Ingenuity pathway analysis (IPA) software, Qiagen).

Further analysis of the DNA microarray data using Ingenuity software (IPA, Qiagen), this time including genes significantly regulated in at least two of the employed ovarian cancer cell lines, not only revealed the chemerin-induced up-regulation of additional IFNα responsive genes (e.g., MX1 and IRF7) but also confirmed a network of induced genes connected with tumor growth inhibition in a more general, not cell-line-specific, manner (Figure 9). IFNα binding to its receptor activates the JAK-STAT pathway, leading to the formation of the ISGF3 transcription factor complex composed of IRF9, STAT1 and STAT2.

### 3.6. Chemerin Effect on IFNα Levels

**Figure 10:**
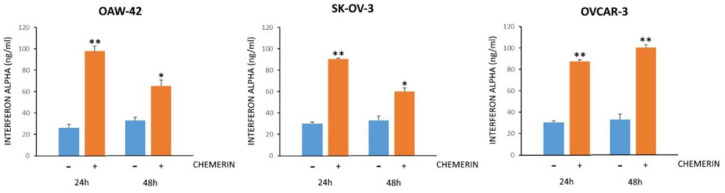
Interferon alpha (IFNα) concentration (ng/mL) in cell culture supernatants of the indicated cell lines after treatment with huChem-157 (400 ng/mL) for 24 or 48 h as assessed via ELISA. * *p* < 0.05; ** *p* < 0.01.

Since pathway analyses strongly suggested IFNα to be the activator of most genes regulated after chemerin treatment in all cell lines tested, but we did not observe any induction of IFNα on the mRNA level itself, we tested the effect of this adipokine on the IFNα protein concentrations in cell culture supernatants using ELISA (DFNAS0, R&D Systems). After 24 and 48 h of chemerin treatment (400 ng/mL), basal IFNα levels significantly increased in the supernatants of all ovarian cancer lines. This observation substantiates the connection between chemerin and the observed activation of IFNα response (Figure 10).

### 3.7. Association of Chemerin-Regulated Genes with Survival of Ovarian Cancer Patients

**Figure 11:**
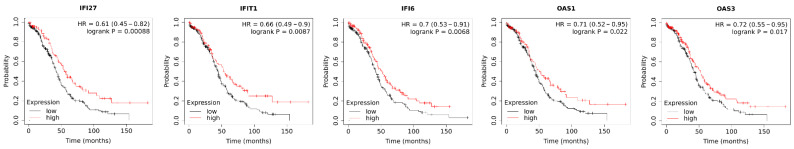
Associations of expression of the indicated interferon response genes markedly up-regulated upon huChem-157 treatment in all ovarian cancer cell lines with overall survival (OS) of 347 ovarian cancer patients. Open-source RNA-seq data in combination with patients´ survival data were provided and examined on the KMplotter website and its online tools at https://kmplot.com/analysis/ (accessed on 20 January 2022) [36].

To assess the clinical relevance of those genes being strongly regulated upon chemerin treatment in vitro, we examined their correlations with patients’ survival using the open-source data and software available at the platform https://kmplot.com/analysis/ (accessed on 15 January 2022) [36]. On the basis of RNA-seq data from 347 ovarian cancer patients, several of these genes were found to be associated with prolonged survival. Positively correlated with overall survival (OS) were the interferon response genes, being strongly induced by chemerin in all three cell lines: IFI27, (HR = 0.61, *p* = 0.0009), IFIT1 (HR = 0.66, *p* = 0.008), IFI6 (HR = 0.7, *p* = 0.007), OAS1 (HR = 0.71, *p* = 0.02) and OAS3 (HR = 0.72, *p* = 0.017) (Figure 11). Among the genes with the most pronounced up-regulation in both OVCAR-3 and SKOV-3 cells, MX1 gene only showed a trend towards prolonged OS (HR = 0.76, *p* = 0.064), just like IFI44L (HR = 0.79, *p* = 0.092). IRF9 expression was not significantly correlated with survival. With regard to genes down-regulated upon chemerin treatment, POGZ, being the only gene with decreased transcript levels in all cell lines employed, showed no correlation to survival of OC patients. In contrast, two down-regulated genes in OVCAR-3 and OAW-42 cells, the USP12 and PTK2, reported to promote tumor cell growth, both exhibited a weak positive correlation with an adverse OS, (HR = 1.17, *p* = 0.018) and (HR = 1.18, *p* = 0.016), respectively.

## 4. Discussion

The results of this study demonstrate an inhibitory effect of bioactive chemerin huChem-157 in vitro on the growth of ovarian cancer cells cultured in 2D or 3D models that was cell-line-specific and was most pronounced in OVCAR-3 cells. On the molecular level, huChem-157 triggered a notable induction of anti-tumoral IFNα response genes and of IFNα protein levels in culture medium with all cell lines tested.

A tumor-suppressing function of chemerin in vivo has been established for many tumor entities, e.g., by recruiting innate immune defenses [15]. High serum chemerin levels were reported to be associated with improved overall survival of patients with adrenocortical carcinoma, acute myeloid leukemia and melanoma [51,52,53]. Expression of chemerin receptor genes CMKLR1, GPR1 or CCRL2 is associated with longer survival in ovarian cancer, breast cancer and non-small-cell lung cancer (NSCLC) [15].

To elucidate whether chemerin would exert direct effects on ovarian tumor cells, which are independent from the activation of immune defenses, and to study the underlying molecular mechanisms, in vitro approaches using defined conditions and models are required. In this in vitro study, we examined the effect of bioactive chemerin huChem-157 on basic cellular functions and on the transcriptome of the ovarian cancer cell lines OVCAR-3, SK-OV-3 and OAW-42. The employed ovarian cancer cell lines were shown to express similar protein levels of chemerin receptor CMKLR1, but they differed in protein expression of GPR1, with the highest levels in OVCAR-3 cells, moderate levels in SK-OV-3 cells and nearly absent expression in OAW-42 cells, suggesting this difference to be one reason for the cell-line specific chemerin sensitivity in terms of growth reduction. Chem-157 reduced OVCAR-3 proliferation in standard 2D adherent culture as well as growth of 3D culture models, decreasing the size of soft-agar colonies and of OVCAR-3 spheroids. SK-OV-3 cells, also expressing GPR1 protein albeit at moderate levels, was the only other cell line with observable chemerin-triggered growth inhibition, resulting in decrease of spheroid size. The fact that OAW-42 cells showed no growth inhibition upon chemerin treatment might result from the nearly absent GPR1 protein expression or other, yet undetermined characteristics of this cell line. All observed in vitro effects of chemerin were triggered only by the higher concentration of 400 ng/mL of recombinant huChem-157, which is equivalent to 100 ng/mL chemerin in human serum. Initially, concentrations of 100 and 400 ng/mL were chosen for our experiments considering a study reporting that 100 ng/mL of recombinant huChem-157 is equivalent to a concentration of 25 ng/mL chemerin using standard ELISA, thus being comparable to usual chemerin serum levels between 25 and 100 ng/mL [44]. However, a weakness of this study is that we did not compare the effect of 100 and 400 ng/mL Chem-157 in each experiment, because in the initial experiment we observed that only the higher concentration showed an effect in 2D cell culture.

Our data demonstrating chemerin-triggered growth inhibition of cancer cells in vitro are in line with several previous studies. Growth-inhibitory effects of chemerin in vitro were reported in studies on neuroblastoma cells, oral squamous cell carcinoma cell lines, breast cancer cells, hepatocellular carcinoma cells and an ovarian granulosa-like tumor cell line [11,28,54,55,56]. However, a recent study reported chemerin-triggered induction of proliferation of an ovarian cancer cell line, but the conflicting data might result from analysis of the cell line HO8910 not included in our study [57].

Our observation that chemerin treatment did not affect cellular migration or invasiveness of the ovarian cancer cell lines tested is in contrast to some in vitro studies, like on the mentioned HO8910 ovarian cancer cell line, on gastric cancer cells [58] or esophageal squamous cancer cells [59], but supports data indicating this adipokine to act in a tissue-specific and cell-line specific manner both in vitro and in vivo (reviewed in [15]).

The fact that chemerin was not able to induce apoptosis in our cell line models is in line with a study reporting similar data on OVCAR-3 cells [60], although it appeared to be in contrast with the notable induction of IFNα response reported to reduce tumor growth at least in part by induction of apoptosis [61]. However, the interferon response gene exhibiting the most prominent transcriptional induction upon chemerin treatment in all cell lines was IFI27 (coding for Interferon alpha-inducible protein 27), which was initially reported to mediate IFN-induced cell death, but recent studies demonstrated that IFI27 is also able to affect cell cycle regulation leading to inhibition of proliferation of cancer cells without apoptosis induction [62,63,64,65,66]. The anti-tumoral role of this gene in ovarian cancer is supported by its association with prolonged survival we found analyzing open-source data. IFI44L (coding for Interferon-induced protein 44-like) is the gene exhibiting the highest up-regulation in chemerin-treated OVCAR-3 cells (23.8-fold) and was also notably induced in SK-OV-3 cells (10.8-fold). IFI44L was reported to block the apoptotic action of IFNα [67] and to down-regulate antiviral IFNα responses [68] Thus, the major up-regulation of IFI27 and particularly IFI44L might explain the observation that apoptosis apparently did not contribute to the noted growth inhibition triggered by chemerin. Additionally, to our knowledge no study exists reporting induction of apoptosis in cancer cell lines in vitro upon chemerin treatment.

The DNA microarray and resulting transcriptome studies we performed to elucidate molecular mechanisms underlying the cellular effects of chemerin treatment revealed a notable up-regulation of a wide set of IFNα responsive genes, for the first time proposing a central function of this cytokine in chemerin signaling. Our data clearly suggest a role of IFNα as mediator of chemerin action, at least in vitro, a hypothesis which is supported by the observation of elevated IFNα protein levels in the cell culture supernatants of all chemerin-treated cell lines

Notably, transcript levels of nine type I interferon response genes were considerably up-regulated upon chemerin treatment in all ovarian cancer cell lines employed. The main upstream key regulators mediating this effect of IFNα/β on activation of responsive genes are transcription factors of the IRF family like IRF9 (interferon regulatory factor 9). IRF9 expression itself was found to be induced at least 5-fold upon chemerin treatment in all cell lines tested. IRF9 is primarily known to play an essential role in anti-viral immunity, but is also involved in central processes of tumor cells, like growth regulation [69]. IRF9 associates with the phosphorylated STAT1:STAT2 dimer to form the transcription factor complex ISGF3, which enters the nucleus [70]. ISGF3 then binds to IFN stimulated response elements (ISREs) present in the regulatory region of interferon responsive genes and activates their transcription. Thus, up-regulation of IRF9, as part of the ISGF3 complex, is an important upstream mechanism leading to the observed induction of interferon response genes. IRF9 has been reported to be the key factor eliciting the antiproliferative effect of IFNα [49]. Regarding IRF9 action in ovarian cancer cells, this protein was also reported to be the key upstream regulator mediating growth-inhibitory effects of IFNα on OVCAR-3 cells [35], which supports our data suggesting a direct link between IRF9 induction and OVCAR-3 growth inhibition.

IFNα has been shown to suppress cancer cell proliferation in vitro [71,72,73]. Our transcriptome analysis showed the ISGF3 target genes IFI27, IFIT1 and IFI6 to exhibit the strongest induction in all cell lines. On the other hand, Ingenuity pathway analysis software (IPA, Qiagen) identified three genes with notably decreased mRNA levels upon chemerin treatment, USP12, CAT and PTK2 (FAK), which exert tumor-promoting effects and stimulate proliferation of cancer cell lines in vitro [74,75,76]. Since the indicated regulation pattern of the genes in this network decreases cancer cell growth, it is suggested to be a major molecular mechanism underlying the observed inhibitory effect of chemerin on 2D and 3D growth of OVCAR-3 cells and on spheroid growth of SK-OV-3 cells. In addition to the induction of IFI27 gene, which is discussed above, IFI6 gene (coding for interferon alpha-inducible protein 6), being more than 10-fold induced upon chemerin treatment in all cell lines, was reported to affect IFNα-triggered regulation of apoptotic cell death in vitro, inhibiting cell death in gastric cancer cells [36] but promoting it in HEK293 cells [30]. Our finding of IFI6 being associated with prolonged overall survival of OC patients and the prediction of Ingenuity pathway analysis software both suggested IFI6 to act as tumor growth inhibitor, which is in line with our experimental data, although it remains unclear whether this protein also has anti-tumoral functions being independent from apoptosis induction. OAS1 (coding for 2′-5′-Oligoadenylate Synthetase 1) is another interferon response gene which was significantly up-regulated on the mRNA level in all cell lines treated with chemerin, with the strongest induction in SK-OV-3 cells. OAS1 gene codes for a dsRNA-activated antiviral enzyme which plays a critical role in cellular innate antiviral response. In addition, it plays a role in other processes such as cell growth and viability and gene regulation. In breast cancer, prostate and cervix carcinoma cells, elevated levels of OAS1 were reported to decrease cell growth [38,39,40]. Although no data on ovarian cancer cells exist, this gene is suggested to be another factor leading to the growth inhibition observed in this study. IFIT1 (coding for interferon-induced protein with tetratricopeptide repeats 1) is another interferon response gene that was significantly induced on the transcript level after chemerin treatment in all ovarian cancer cell lines tested, with the strongest increase in OVCAR-3 cells. IFIT1 primarily acts as a sensor of viral single-stranded RNAs and inhibits expression of viral messenger RNAs. It also plays a key role in growth suppression and induction of cell death in cancer cells of different origin [77]. In glioblastoma, IFIT1 is overexpressed in more than 80% of the cases, associated with a favorable outcome and long progression-free survival [78]. Our in silico analyses showed ITIT1 to be also associated with beneficial OS of ovarian cancer patients. Since IFIT1 is able to reduce tumor cell growth both in vitro and in vivo, it is tempting to speculate that the strong chemerin effect on IFIT1 expression present in OVCAR-3 cells (16-fold up-regulation) could be another mechanism underlying the decreased growth of this cell line.

As mentioned above, several genes down-regulated upon chemerin treatment in at least two cell lines including OVCAR-3, are known to promote tumor growth (Figure 5). Knockdown of USP12 (Ubiquitin-specific protease 12) has been reported to inhibit proliferation of hepatocellular carcinoma cell lines via p38/MAPK pathway [74]. In contrast, overexpression of USP12 by super-enhancers led to oncogenic effects like elevated growth and increased viability of different epithelial cancer cell lines [79]. Thus, it is tempting to speculate that chemerin-triggered decrease of USP12 expression might be involved in the reduced growth particularly of OVCAR-3 cells observed in this study. PTK2, protein tyrosine kinase 2, better known as FAK (focal adhesion kinase), plays a key role in focal cell adhesion and is a substrate of oncogene *v-src* [80]. This gene exhibited significantly lower mRNA levels after the chemerin treatment of OVCAR-3 and OAW-42 cells. PTK2 is known to be overexpressed in cancer including 86% of serous ovarian cancer cases, being strongly associated with a poor prognosis, suggesting a tumor-promoting role of this gene in ovarian cancer [81,82]. Decreased PTK2 expression has been reported to have a growth-inhibitory impact [83], and its knockdown in ovarian cancer cell lines reduced cell viability and anchorage-independent growth [84]. Thus, the down-regulation of PTK2 is another candidate mechanism for underlying the chemerin effect on OVCAR-3 cells. Finally, the gene exhibiting the most significant mRNA down-regulation after chemerin treatment in OVCAR-3 cells (4.47-fold) was TNFRSF11B, coding for TNF receptor superfamily member 11b, also called osteoprotegerin (OPG). Beyond its established role in bone metabolism and metastasis, the expression of this receptor has been reported to enhance the proliferation of breast cancer cells in vivo and in vitro [85], and similar effects were reported on leukemic cells [86]. One of the proposed oncogenic mechanisms of TNFRSF11B is counteracting the anti-tumoral activity of TRAIL [87]; although its in vitro effects on cancer cell lines have to be further investigated, the present data suggest that the observed down-regulation of TNFRSF11B could possibly contribute to the observed effects of chemerin on OVCAR-3 cells.

Finally, the mechanism underlying the chemerin-triggered increase of IFNα levels in culture medium is suggested to be the reported positive feedback loop involving the IRF9/ISGF3-triggered activation of IFNα expression via IRF7, also being up-regulated in this study [88,89,90].

## 5. Conclusions

The results of this study demonstrate the growth-inhibitory actions of chemerin on ovarian cancer cell lines in vitro. The main molecular mechanism underlying this differentially pronounced effect was a notable induction of anti-tumoral IFNα-response genes, which was partially accompanied by down-regulation of tumor-promoting genes. Our data for the first time show a role of IFNα as a mediator of chemerin action, further corroborated by an increase of secreted IFNα protein levels upon treatment with this adipokine, suggested to result from an ISGF3-mediated positive feedback loop.

## Data Availability

Data will be provided on request.

## Supplementary Materials

The following supporting information can be downloaded at: https://www.mdpi.com/article/10.3390/cancers14174108/s1, Figure S1: Flow cytometric analysis of cell death of the indicated ovarian cancer cell line after 48 h of treatment with 400 ng/mL huChem-157 (Guava^®^ Muse^®^ Cell Analyzer, Luminex, Austin, TX, USA), using the Muse^®^ Annexin V & Dead Cell Kit. As a positive control, a combination of TNFα and BV6 (the latter being an antagonist of IAP inhibitor of apoptosis proteins) was used. Figure S2: Microphotographs of the indicated ovarian cell lines, not showing any change of cell morphology after treatment with chemerin, in contrast to the positive control treatment resulting in visible cell damage. Table S1: Genes exhibiting the strongest change of transcript levels 48 h after chemerin treatment in the indicated ovarian cancer cell lines, as assessed by transcriptome analysis by means of Affymetrix Human Clariom S arrays. File S1. Full pictures of the Western blots for Figure 1 and Figure 6.
