# Supplementary material for: Anti-Tumoral Effect of Chemerin on Ovarian Cancer Cell Lines Mediated by Activation of Interferon Alpha Response
Source: Cancers (Basel). 2022 Aug 25;14(17):4108. doi: 10.3390/cancers14174108 (PMC9454566; doi:10.3390/cancers14174108)
Supplement: Supplementary file 1 [file cancers-14-04108-s001.zip › Supplemental Table S1.pdf]

Table S1 (a) Genes exhibiting the strongest increase of transcript levels 48 h after chemerin treatment in the indicated ovarian cancer cell lines, as assessed by Affymetrix Human Clariom S arrays.

**OVCAR-3 chemerin up-regulated genes (top 10)**		
Gene	*Gene name*	*-fold change*
IFI44L	interferon-induced protein 44-like	23,82
IFI27	interferon, alpha-inducible protein 27	22,20
IFIT1	interferon-induced protein with tetratricopeptide repeats 1	16,49
IFI6	interferon, alpha-inducible protein 6	14,09
BST2	bone marrow stromal cell antigen 2	9,29
MX1	MX dynamin-like GTPase 1	9,01
IRF9	interferon regulatory factor 9	5,82
OAS1	2-5-oligoadenylate synthetase 1	5,54
SAMD9L	sterile alpha motif domain containing 9-like	3,69
CNTNAP3	Salzman2013 ANNOTATED, CDS, best transcript NM_001201380	3,64
**OAW-42 chemerin up-regulated genes (top 10)**		
Gene	*Gene name*	*-fold change*
IFI6	interferon, alpha-inducible protein 6	14,96
OAS1	2-5-oligoadenylate synthetase 1	8,23
IRF9	interferon regulatory factor 9	5,83
EPS8	epidermal growth factor receptor pathway substrate 8	4,43
TINAGL1	tubulointerstitial nephritis antigen-like 1	3,98
IFI27	interferon, alpha-inducible protein 27	3,92
ADA	adenosine deaminase	3,63
IFIT1	interferon-induced protein with tetratricopeptide repeats 1	3,63
BST2	bone marrow stromal cell antigen 2	3,54
IFIH1	interferon induced, with helicase C domain 1	3,39
**SK-OV-3 chemerin up-regulated genes (top 10)**		
Gene	*Gene name*	*-fold change*
IFI27	interferon, alpha-inducible protein 27	68,42
OAS1	2-5-oligoadenylate synthetase 1	44,46
OAS2	2-5-oligoadenylate synthetase 2	39,94
XAF1	XIAP associated factor 1	38,62
IFI6	interferon, alpha-inducible protein 6	33,44
MX1	MX dynamin-like GTPase 1	30,31
IRF9	interferon regulatory factor 9	13,37
IFI44L	interferon-induced protein 44-like	10,83
IFIT1	interferon-induced protein with tetratricopeptide repeats 1	7,85
OAS3	2-5-oligoadenylate synthetase 3	6,95

Table S1 (b) Genes exhibiting the strongest reduction of transcript levels 48 h after chemerin treatment in the indicated ovarian cancer cell lines, as assessed by Affymetrix Human Clariom S arrays.

OVCAR-3 chemerin down-regulated genes (top 10)		
Gene symbol	*Gene name*	*-fold change*
ANKRD18B	ankyrin repeat domain 18B	-3,00
NDUFA8	Transcript Identified by AceView, Entrez Gene ID(s) 4702	-3,03
ZNF200	Transcript Identified by AceView, Entrez Gene ID(s) 7752	-3,06
NR2E1	nuclear receptor subfamily 2, group E, member 1	-3,07
ISPD	isoprenoid synthase domain containing	-3,15
FLJ45513	uncharacterized LOC729220	-3,40
TMEM155	transmembrane protein 155	-3,54
ZNF730	zinc finger protein 730	-3,59
AMTN	amelotin	-3,63
TNFRSF11B	tumor necrosis factor receptor superfamily, member 11b	-4,47
OAW-42 chemerin down-regulated genes (top 10)		
Gene symbol	*Gene name*	*-fold change*
SLC38A9	Transcript Identified by AceView, Entrez Gene ID(s) 153129	-4,28
POGZ	Transcript Identified by AceView, Entrez Gene ID(s) 23126	-4,45
EXOC4	exocyst complex component 4	-4,60
GPCPD1	Transcript Identified by AceView, Entrez Gene ID(s) 56261	-5,08
EXT1	Jeck2013 ALT_ACCEPTOR, ALT_DONOR, coding, INTERNAL, intronic best	-5,62
PTBP2	Transcript Identified by AceView, Entrez Gene ID(s) 58155	-5,98
WWP1	Transcript Identified by AceView, Entrez Gene ID(s) 11059	-6,07
EXT1	Jeck2013 ALT_ACCEPTOR, ALT_DONOR, coding, INTERNAL, intronic best	-6,15
PPM1B	Memczak2013 ANTISENSE, coding, INTERNAL, intronic best transcript	-6,87
OCLM	Transcript Identified by AceView, Entrez Gene ID(s) 10896	-7,01
SK-OV-3 chemerin down-regulated genes (top 10)		
Gene symbol	*Gene name*	*-fold change*
FILIP1	filamin A interacting protein 1	-2,63
SPATA5	Transcript Identified by AceView, Entrez Gene ID(s) 166378	-2,70
VAPA	Memczak2013, coding, INTERNAL, intronic best transcript NM_003574	-2,76
LRRC28	Transcript Identified by AceView, Entrez Gene ID(s) 123355	-2,90
GNGT1	(G protein), gamma transducing activity polypeptide 1	-2,98
PGR	progesterone receptor	-3,03
UNC93A	unc-93 homolog A (C. elegans)	-3,19
PHYHIPL	phytanoyl-CoA 2-hydroxylase interacting protein-like	-3,32
PLEKHG4B	Zhang2013 coding, INTERNAL, intronic best transcript NM_052909	-3,42
PPM1B	Memczak2013 ANTISENSE, coding, intronic best transcript NM_001033556	-13,27
