# Supplementary figures and images for: Anti-Tumoral Effect of Chemerin on Ovarian Cancer Cell Lines Mediated by Activation of Interferon Alpha Response
Source: Cancers (Basel). 2022 Aug 25;14(17):4108. doi: 10.3390/cancers14174108 (PMC9454566; doi:10.3390/cancers14174108)

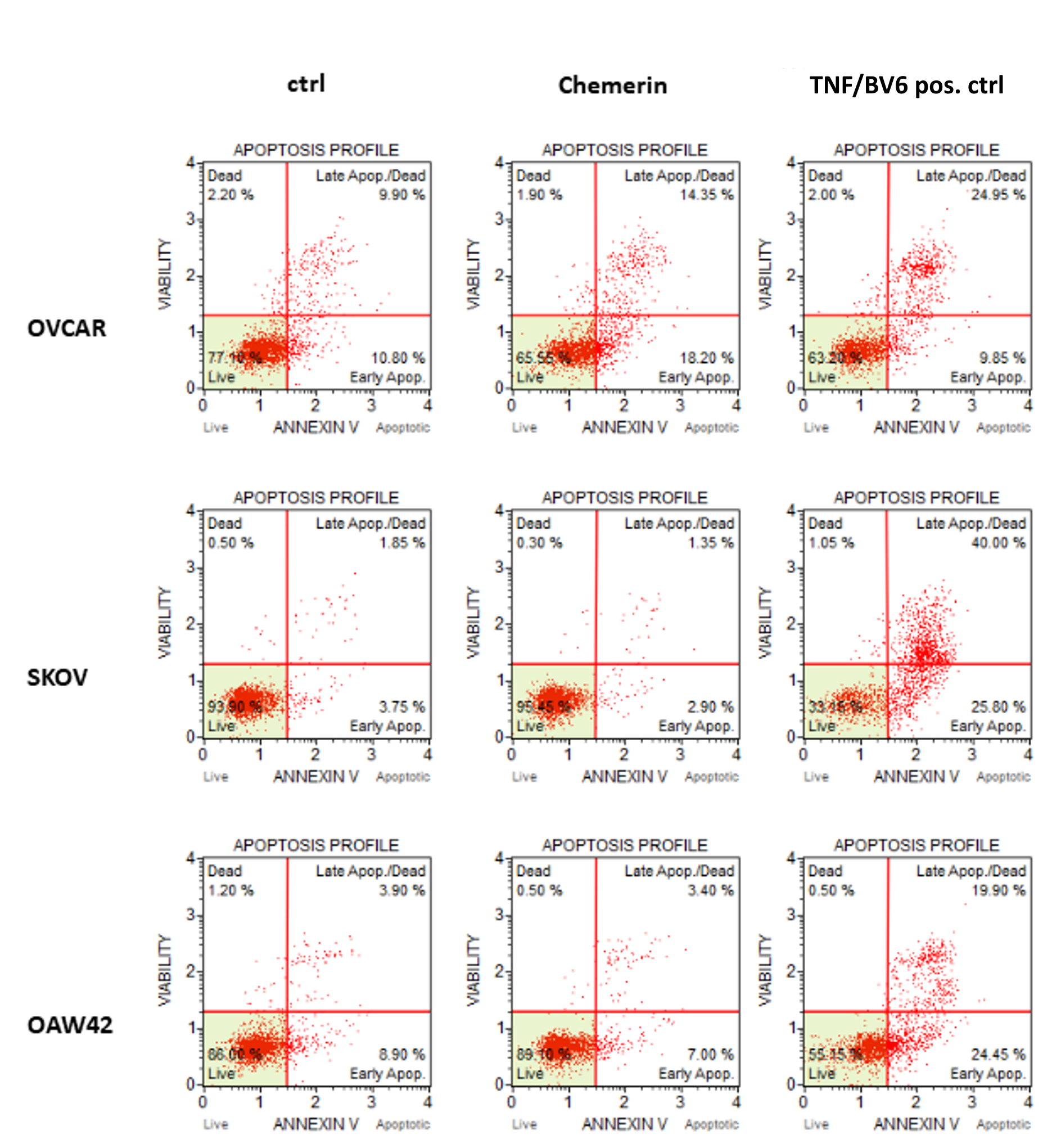

Supplement: Supplementary file 1 [file cancers-14-04108-s001.zip › Supplemental Fig. S1.tiff]

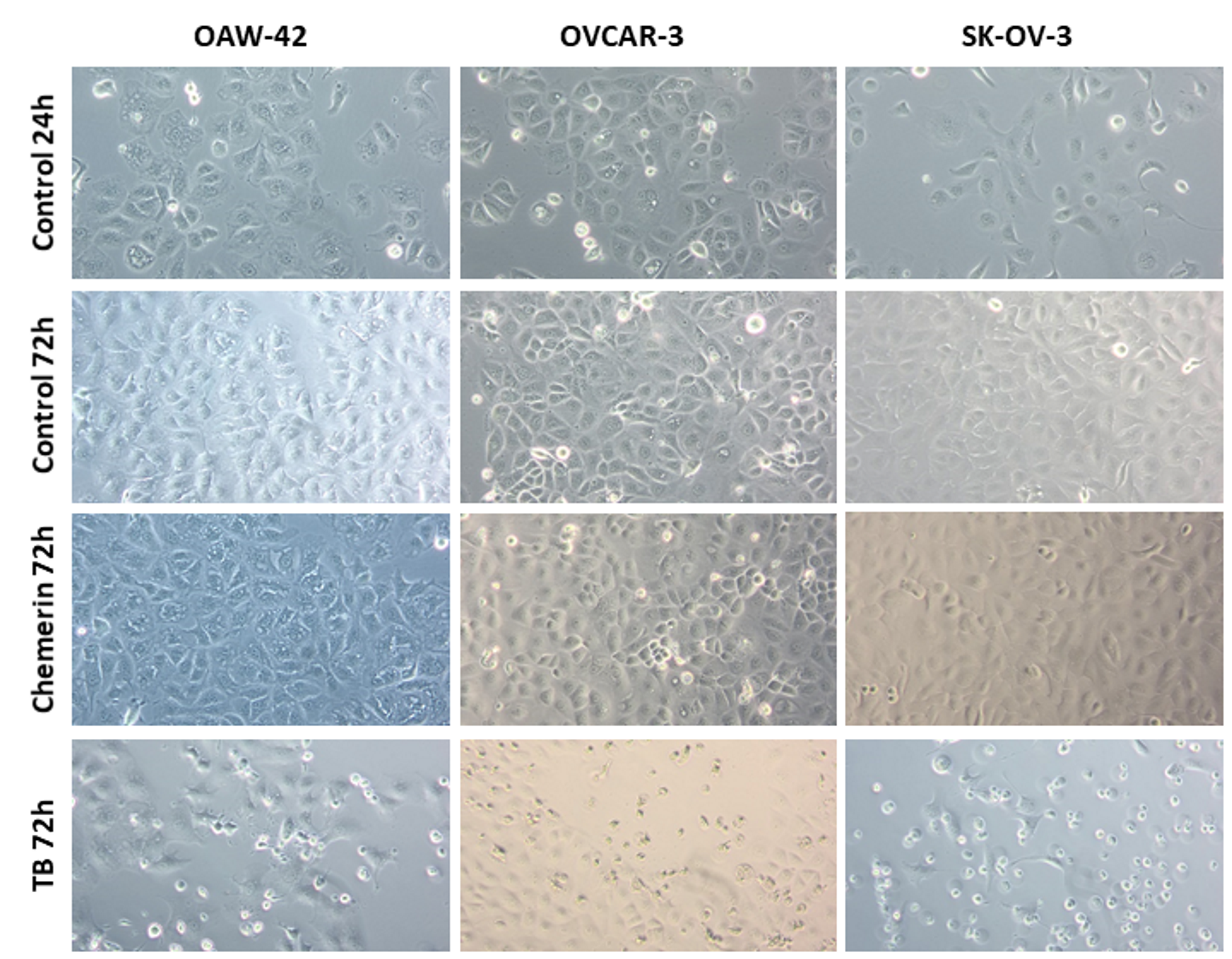

Supplement: Supplementary file 1 [file cancers-14-04108-s001.zip › Supplemental Fig. S2.tiff]
